# Crocin alleviates the local paw edema induced by histamine in rats

**Published:** 2012

**Authors:** Esmaeal Tamaddonfard, Amir Abbas Farshid, Leila Hosseini

**Affiliations:** 1 *Department of Basic Sciences, Faculty of Veterinary Medicine, Urmia University, Urmia, I. R. Iran*; 2 *Department of Pathobiology, Faculty of Veterinary Medicine, Urmia University, Urmia, I. R. Iran*

**Keywords:** Crocin, Chlorpheniramine, Histamine, Paw edema, Rats

## Abstract

**Objective: **Crocin, as an active constituent of saffron, has many biological functions including antioxidant and anti-inflammatory activities. The present study was aimed to investigate the effects of crocin and chlorpheniramine on local edema induced by histamine.

**Materials and Methods: **Local edema was induced by subcutaneous injection of histamine (100 μl, 0.1%) in ventral surface of right hind paw. The thickness of paw was measured at 1 h before and 1, 2, 3 h after injection of histamine, using a fine caliper. The number of neutrophils in paw tissue sections was counted 3 h after intraplantar injection of histamine.

**Results: **Intraperitoneal injection of crocin at doses of 100 and 200 mg/kg and chlorpheniramine at a dose of 10 mg/kg significantly (p<0.05) decreased both paw thickness and infiltration of neutrophils in paw tissues. In a combined treatment, intraperitoneal injection of an effective dose of crocin (100 mg/kg) with an ineffective dose of chlorpheniramine (2.5 mg/kg) produced a more documented response in comparison with crocin (100 mg/kg) and chlorpheniramine (2.5 mg/kg) used alone.

**Conclusion: **The results suggested that both crocin and chlorpheniramine suppressed histamine-induced local paw edema. Moreover, histamine H_1_ receptors function may be affected by crocin.

## Introduction

Crocin is a digentiobiosyl all-*trans*-crocetim (8,8′-di-apocarotene-8,8′-dioic acid) ester that is the major yellow pigment in gardenia yellow and saffron, which are extracts of *Gardenia jasminoides* fruits and *Crocus sativus* stigmas, respectively (Aung et al., 2007 ▶; Lee et al., 2007 ▶). Saffron is used in folk medicine for various purposes such as an antispasmodic, nerve sedative, eupeptic, anticatarrhal, carminative, diaphoteric, stomachic, and aphrodisiac substance, and also as an emmenagogue and expectorant (Schmidt et al., 2007 ▶). Saffron contains carotenoid pigments called tricrocin, bicrocin, and crocin, a bitter glycoside called picocrocin, and the volatile, aromatic substance, safranal (Ochiai et al., 2007 ▶; Rios et al., 1996 ▶).

 In modern pharmacological studies, saffron and its constituents including crocin and safranal, have demonstrated anticonvalsant, antidepressant, antioxidant, and antitumour properties (Abdullaev, 2002 ▶; Hosseinzadeh and Khosravani 2002 ▶; Hosseinzadeh et al., 2004 ▶; Zheng et al., 2007 ▶). Crocin inhibited xylene-induced ear edema and carrageenan-induced paw edema in rats (Xu et al., 2009 ▶). Moreover, in isoproterenol-induced cardiotoxicity, crocin suppressed edema as well as inflammatory cells infiltration in myocardium (Goyal et al., 2010 ▶). 

Histamine is a biogenic amine and through its specific membrane receptors, H_1_, H_2_, H_3_, and H_4_, plays an important role in physiological and pathological processes such as gastric acid secretion, smooth muscle contraction, neurotransmission, immunomodulation, angiogenesis, and allergic disorders (Parsons and Ganellin, 2006 ▶; Tripathi et al., 2010 ▶). Histamine affects the local inflammatory responses by activation of vasodilation, vascular permeability, edema formation, polymorphonuclear leukocyte infiltration, and cytokine release (Bilici et al., 2001 ▶; Chimona et al., 2007 ▶; Ozbakis-Dengiz et al., 2007 ▶; Kalokasidis et al., 2009 ▶; Farshid et al., 2011 ▶).

Histamine-induced inflammation has been widely used to explore the anti-inflammatory effects of some medicinal plants. The aqueous and methanol extract of the dried latex of *Calotropis procera* and the leaf extract of *Garcinia gardneriniana* reduced paw edema induced by histamine, serotonin, and prostaglandin E_2_ (Arya and Kumar, 2005 ▶; Castardo et al., 2008 ▶). 

The effects of crocin on histamine-induced paw edema, to the best of our knowledge, have not been studied before. In the present study, the effect of crocin was investigated on paw edema induced by intra-plantar injection of histamine in rats. In addition, to identify the mechanism that possibly mediates the effect of crocin on local paw edema induced by histamine, the contribution of histamine H_1_ receptors was assessed using chlorpheniramine, a histamine H_1_ receptor antagonist, with and without crocin. 

## Materials and Methods

**Animals**

Healthy adult male Wistar rats, weighing 210 – 230 g were used in this study. Rats were maintained in polyethylene cages with food and water available *ad libitum*, in a laboratory with controlled ambient temperature (23º±0.5°C) and under a 12:12 h light-dark cycle (lights on from 07:00 h). Six rats were used in each experiment. The experimental protocol was approved by the Veterinary Ethics Committee of the Faculty of Veterinary Medicine of Urmia University. 

**Drugs**

The following drugs were administered: histamine, crocin, and chlorpheniramine. The drugs were purchased from Sigma–Aldrich (Chemical Co., Inc., St Louis, MO, USA). The drugs were dissolved in sterile normal saline. 

**Treatment groups**

Rats were divided into following seven groups of six animals each. Group I was treated with intraperitoneal injection of normal saline followed by intraplantar injection of 0.1% histamine. In groups II, III and IV, intraplantar injections of histamine were performed after intraperitoneal injection of crocin at doses of 50, 100 and 200 mg/kg. Groups V and VI were treated with intraperitoneal injection of chlorpheniramine at doses of 2.5 and 10 mg/kg before intraplantar injection of histamine. Group VII received intraperitoneal injection of crocin (100 mg/kg) plus chlorpheniramine (2.5 mg/kg) before intraplantar injection of histamine. Crocin and chlorpheniramine were administered 30 and 20 min before intraplantar injection of histamine, respectively. The drug doses used here were selected according to the investigations in which the used doses of crocin and chlorpheniramine were 25-200 mg/kg and 3-10 mg/kg, respectively (Kohno et al., 1987 ▶; Shivkar and Kumar, 2003 ▶; Tamaddonfard and Hamzeh-Gooshchi, 2010 ▶). In the present study, we used chlorpheniramine as a reference drug according to the previous studies (Arya and Kumar, 2005 ▶; Boskabady et al., 2010 ▶; Boskabady et al., 2011 ▶). 

**Induction of paw edema**

For induction of paw edema, each rat was subcutaneously injected with 100 µl histamine (0.1%) in the ventral surface of the right hind paw using a 29-gauge injection needle and was returned to its cage (Bilici et al., 2001 ▶; Ozbakis-Dengiz et al., 2007 ▶; Farshid et al., 2011 ▶). The magnitude of paw edema was assessed by measuring dorsal-plantar paw thickness with a fine caliper (Farshid et al., 2010 ▶ and 2011 ▶), at 1 h before and 1, 2 and 3 h after histamine injection (Farshid et al., 2011 ▶). Edema was expressed as the increase in paw thickness (mm) after histamine injection relative to the pre-injection value for each animal (Farshid et al., 2010 ▶ and 2011 ▶). 

**Histopathological evaluation**

For histopathological evaluation of paw tissues, the animals were euthanized by decapitation 3 h after histamine injection, and their paw tissues were collected for histopathological investigation. The specimens were fixed in 10% buffer formal saline and routinely processed for paraffin embedding. For each sample, 4-5 µm thick sections were cut and stained with hematoxylin-eosin, to evaluate the acute inflammation. Neutrophils were counted by special morphometric lens in 0.25 mm^2 ^microscopic field, from 10 different areas of the sections and the mean values were calculated. The final number of neutrophils was expressed as the mean of the number counted in six animals per group (Farshid et al., 2010 ▶ and 2011 ▶) 

**Statistical analysis**

Reported values are the mean±SEM. Statistical analysis was performed by repeated measure analysis of variance (ANOVA) and Duncan's test for the data obtained from the paw edema. Data obtained from the neutrophil infiltration were analyzed by one-way analysis of variance (ANOVA) followed by Duncan's test. The significant level was expressed as p<0.05. 

## Results

**Figure 1:**
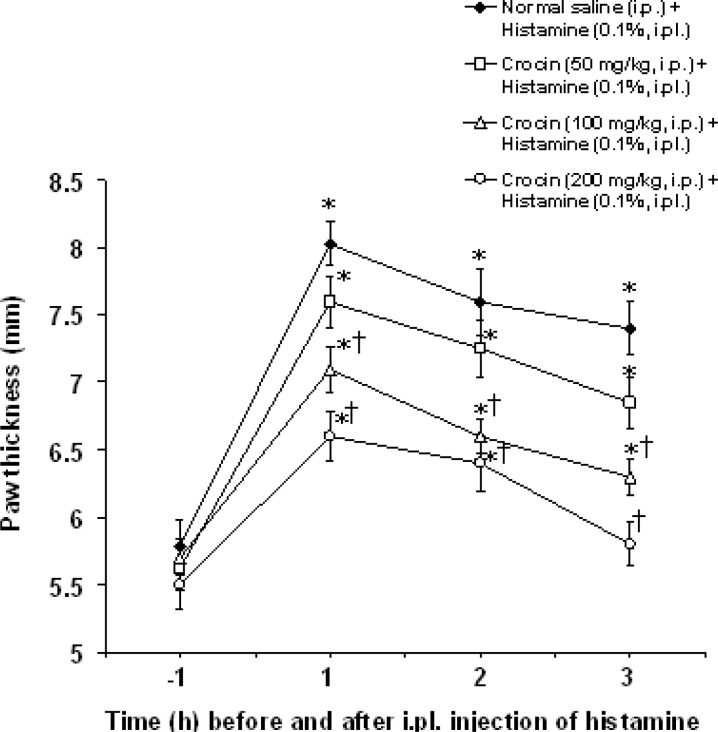
Effect of crocin on paw thickness induced by intraplantar injection of histamine in rats. All values are expressed as mean±SEM (n=6). Statistical comparisons among groups were carried out by factorial ANOVA followed by Duncanʹs tests. *p<0.05 in comparison with 1 h before histamine injection. ^†^p<0.05 in comparison with normal saline + histamine group. ip: intraperitoneal, ipl: intraplantar.

Intraplantar injection of histamine evoked a local edema with maximal rate detected within 1 h after injection and thereafter declined to the end of the experiment. Intraperitoneal injections of crocin at doses of 100 and 200 mg/kg, but not at a dose of 50 mg/kg, significantly decreased 1, 2 and 3 h paw thickness induced by histamine (F_(3,80)_ = 23.636, p<0.05) (Figure 1). 

**Figure 2:**
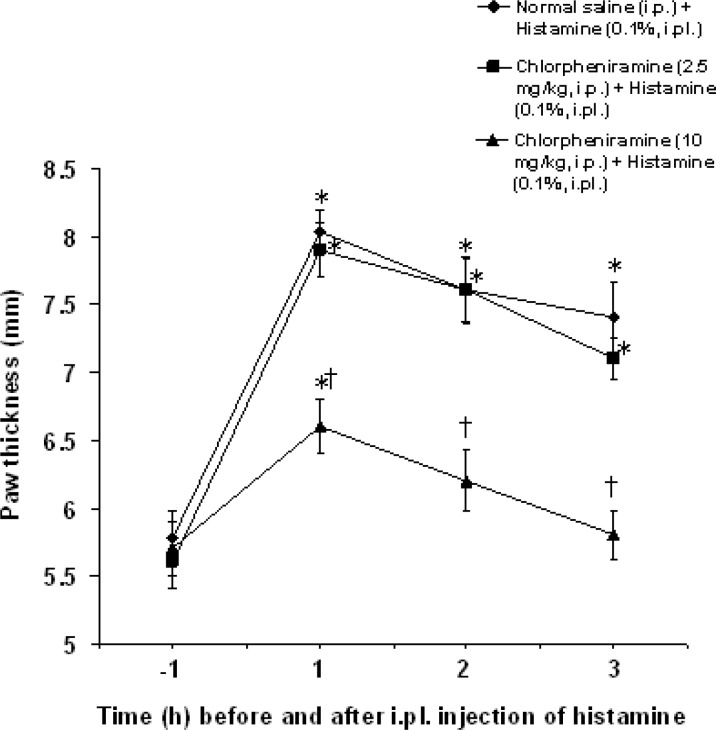
Effect of chlorpheniramine on paw thickness induced by intraplantar injection of histamine in rats. All values are expressed as mean ±SEM (n=6). Statistical comparisons among groups were carried out by factorial ANOVA followed by Duncanʹs tests. *p<0.05 in comparison with 1 h before histamine injection. ^†^p<0.05 in comparison with normal saline+histamine and chlorpheniramine (2.5 mg/kg) + histamine groups. ip: intraperitoneal, ipl: intraplantar.

Intraperitoneal injection of chlorpheniramine at a dose of 10 mg/kg, but not at a dose of 2.5 mg/kg, significantly decreased 1, 2 and 3 h paw thickness induced by histamine (F_(2,60)_ = 32.222, p<0.05) (Figure 2). 

**Figure 3:**
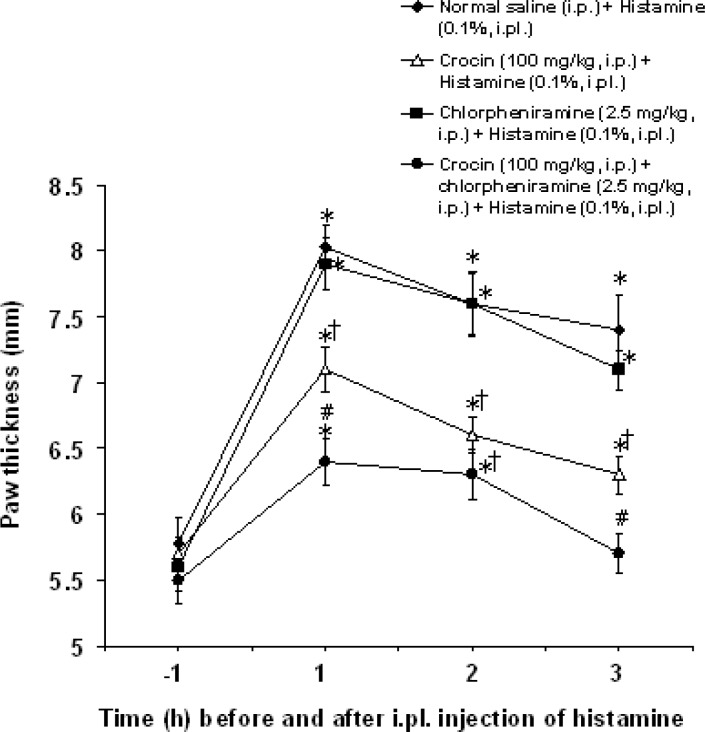
Effect of co-administration of crocin with chlorpheniramine on paw thickness induced by intraplantar injection of histamine in rats. All values are mean±SEM (n=6). Statistical comparisons among groups were carried out by factorial ANOVA followed by Duncanʹs tests. *p<0.05 in comparison with 1 h before histamine injection. ^†^p<0.05 in comparison with normal saline+histamine and chlorpheniramine (2.5 mg/kg) + histamine groups. ^‡^p<0.05 in comparison with crocin (100 mg/kg) +histamine and chlorpheniramine (2.5 mg/kg) +histamine groups. ip: intraperitoneal, ipl: intraplantar.

Intraperitoneal co-administration of an effective dose of crocin (100 mg/kg) with an ineffective dose of chlorpheniramine (2.5 mg/kg) decreased the paw thickness as compared with crocin (100 mg/kg) and chlorpheniramine (2.5 mg/kg) used alone (F_(3,80)_ =17.458, p<0.05) (Figure 3).

**Figure 4:**
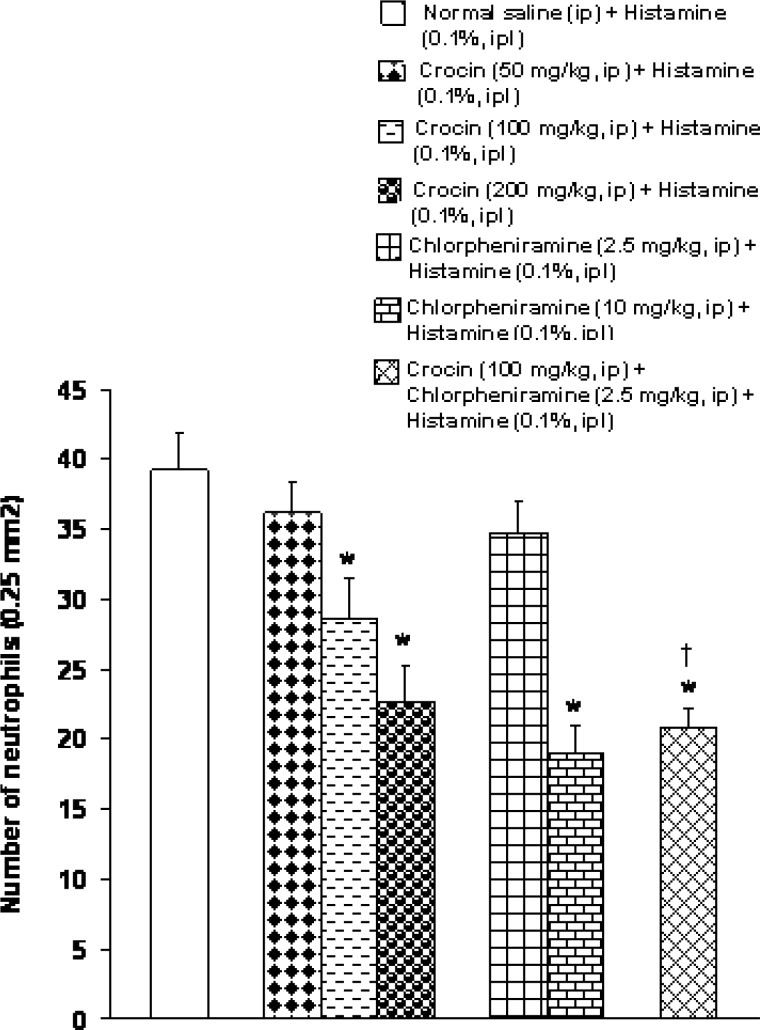
Effects of separate and combined treatments with crocin and chlorpheniramine on the number of neutrophils in paw tissues in rats. All values are mean±SEM (n=6). Statistical comparisons among groups were carried out by one way ANOVA followed by Duncanʹs tests. *p<0.05 in comparison with normal saline+histamine group. ^†^p< 0.05 in comparison with chlorpheniramine (2.5 mg/kg) +histamine and criocin (100 mg/kg) +histamine groups. ip: intraperitoneal, ipl: intraplantar.

**Figure 5:**
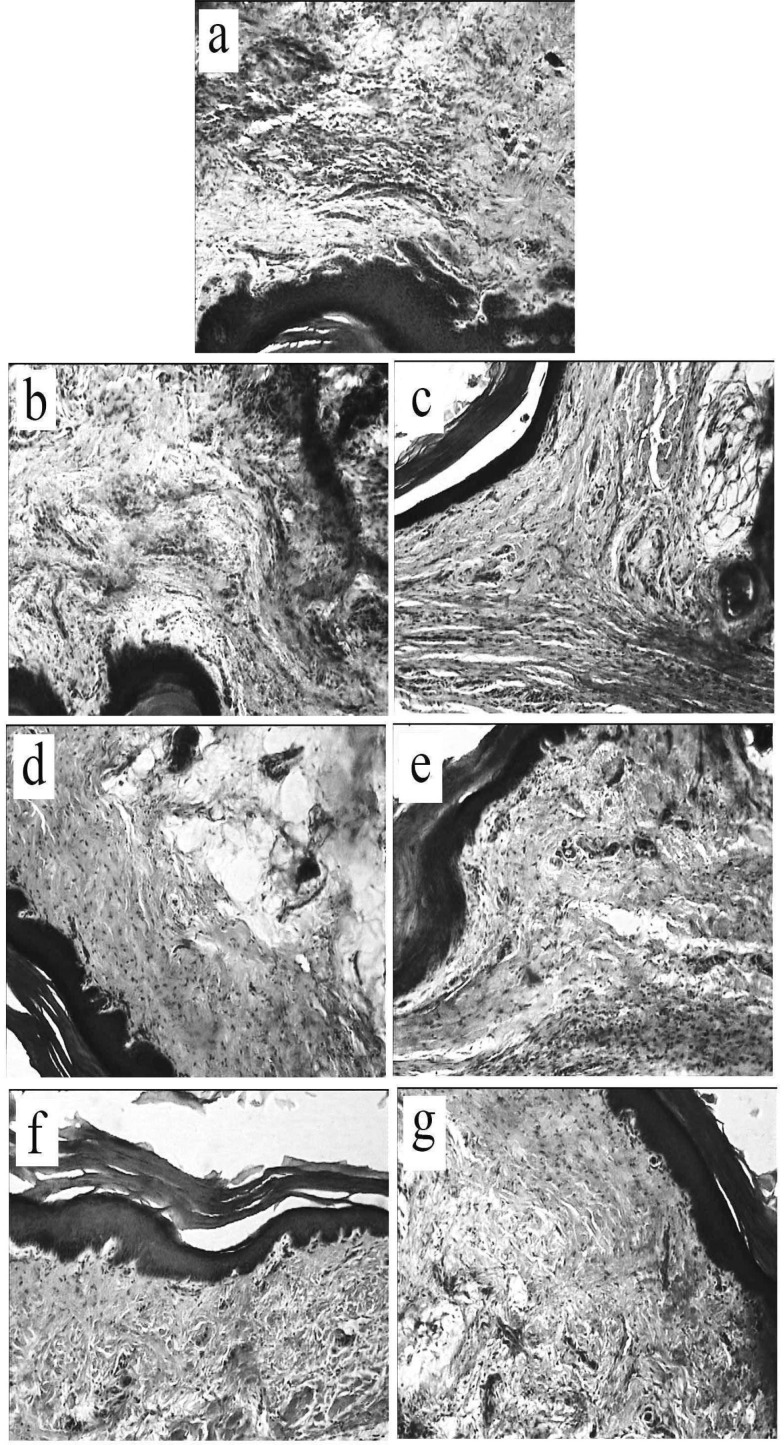
Effects of separate and combined treatments with crocin and chlorpheniramine on neutrophil infiltration induced by histamine in rat paw tissues. Animals were treated with: (a) normal saline + histamine, (b) crocin (50 mg/kg) +histamine, (c) crocin (100 mg/kg) + histamine, (d) crocin (200 mg/kg) +histamine, (e) chlorpheniramine (2.5 mg/kg) +histamine, (f) chlorpheniramine (10 mg/kg) + histamine, (g) crocin (100 mg/kg) + chlorpheniramine (2.5 mg/kg) +histamine. Extensive neutrophil infiltrations are seen in a, b, and e. Moderate neutrophil infiltrations are seen in c, d, f, and g (H&E × 100).

Histopathologically, congestion, edema, haemorrhages, and leukocytic infiltration, mainly neutrophils, were observed in the inflamed area. As presented in Figure 4 and showed in Figure 5a, the number of neutrophils was highest (39.2±2.7) in the intraplantar injected (control) group. Intraperitoneal injection of crocin at a dose of 50 mg/kg had no significant effect, but at doses of 100 and 200 mg/kg, crocin significantly decreased the number of neutrophils in the inflamed area (F_(3,20)_ =11.336, p <0.05) (Figures 4, 5b, 5c, and 5d). Intraperitoneal injection of chlorpheniramine at a dose of 10 mg/kg, but not at a dose of 2.5 mg/kg, significantly decreased the neutrophil infiltration (F_(2,15)_ =22.104, p <0.05) (Figures 4, 5e, and 5f). Intraperitoneal co-administration of an effective dose of crocin (100 mg/kg) with an ineffective dose of chlorpheniramine (2.5 mg/kg) decreased the number of neutrophils as compared with crocin (100 mg/kg) and chlorpheniramine (2.5 mg/kg) used alone (F_(3,20)_ =23.729, p<0.05) (Figures 4 and 5g).

## Discussion

In this study, subcutaneous injection of histamine in the ventral surface of hind paw produced paw edema as well as neutrophil infiltration in the paw tissue. In addition, pretreatment with chlorpheniramine suppressed histamine-induced paw inflammatory responses. Histamine-induced inflammation has been well established as a valid model to study paw edema and neutrophil infiltration in paw tissue after inflammatory states (Bilici et al., 2001 ▶; Ozbakis-Dengiz et al., 2007 ▶; Farshid et al., 2011 ▶). Several reports have confirmed that histamine alone and in participation with chemoattractants such as platelet activating factor, interleukin 8, and leukotriene B_4_ involve in the regulation of neutrophil recruitment (Bilici et al., 2001 ▶; Chimona et al., 2007 ▶; Ozbakis-Dengiz et al., 2007 ▶; Kalokasidis et al., 2009 ▶; Farshid et al., 2011 ▶; Perretti et al., 1994 ▶).

Histamine H_1_ receptors are involved in mediating the inflammation induced by various inflammatory agents. Oral administration of chlorpheniramine suppressed histamine-induced paw edema in rats (Arya and Kumar, 2005 ▶). Moreover, intravenous injection of chlorpheniramine inhibited the edema response induced by subcutaneous injection of substance P, a mediator of inflammation (Gilligan et al., 1994 ▶), in mouse ear (Inoue et al., 1996 ▶). Paw edema induced by subplantar injection of zymogen was inhibited by intraperitoneal injections and oral administrations of clemastine, cyproheptadine, cetirizine, loratadine, and terfenadine (histamine H_1_ receptor antagonists) in rats (Blazso and Gabor, 1997 ▶). In addition, oral administration of desloratadine and levocetirizine (histamine H_1_ receptor antagonists) for 3 days reduced the oedema, vascular dilation and congestion induced by transtympanically injection of histamine on the right middle ear mucosa in rabbits (Chimona et al., 2008 ▶). 

The results of present study indicated that crocin suppressed the paw edema and neutrophil recruitment induced by histamine. Moreover, a potentiation effect was observed between crocin and chlorpheniramine when an effective dose of crocin was used with an ineffective dose of chlorpheniramine. These indicate that crocin produces an anti-edematous effect on histamine-induced local paw edema. In addition, crocin may affect the histamine H_1_ receptors function. To our knowledge, this is the first report that provided an evidence of anti-edematous effect of crocin on histamine-induced paw edema in rats. It is well known that histamine is synthesized and released by different cells including basophils, mast cells, platelets, histaminergic neurons, lymphocytes, and entrochromaffin cells (Criado et al., 2010 ▶).

Crocin is a major constituent of saffron (Rios et al., 1996 ▶), and the inhibitory effect of a water extract of a mixture of eight herbs including chamomiles, saffron, anise, fennel, caraway, licorice, cardamom, and black seed was reported on compound 48/80- and IgE/anti-IgE-induced histamine release from peritoneal mast cells (Haggag et al., 2003 ▶). Crocin may affect the function of histamine H_1_ receptors, because the inhibitory effect of aqueous-ethalonic extract of saffron has been reported on the function of histamine H_1_ receptors in the guinea pig tracheal chains (Boskabady et al., 2010 ▶). 

Besides, recently the inhibitory effect of saffron on the function of histamine H_1_ receptors has been attributed to safranal, another constituent found in saffron (Boskabady et al., 2011 ▶). In other inflammatory states, crocin exerts beneficial effects. Oral administration of crocin inhibited the xylene-induced ear edema in mice and carrageenan-induced paw edema and reduced the rise in hind paw prostaglandin E_2_ level in carrageenan-induced paw edema in rats (Xu et al., 2009 ▶). In the isoproterenol-induced cardiotoxicity, the preventive effect of chronic oral administration of crocin on myocardial necrosis, edema, and inflammation has been attributed to the recovery effects of crocin on cardiac levels of CK-MB, LDH, MDA, catalase, SOD, and GSH in rats (Goyal et al., 2010 ▶). It seems that crocin exerts its anti-inflammatory effect through several mechanisms. 

In conclusion, crocin produced an anti-edematogenic effect in histamine-induced local paw edema in rats. A potentiation effect was observed between crocin and chlorpheniramine. Histamine H_1_ receptors function may be influenced by crocin. 
